# Comprehensive care for people with sexually transmitted infections

**DOI:** 10.1590/0037-8682-954-2020

**Published:** 2021-05-17

**Authors:** Taís Freire Galvão, Carlos Henrique Nery Costa, Leila Posenato Garcia

**Affiliations:** 1 Universidade Estadual de Campinas, Faculdade de Ciências Farmacêuticas, Campinas, SP, Brasil.; 2 Universidade Federal do Piauí, Centro de Inteligência em Agravos Tropicais Emergentes e Negligenciados (CIATEN), Departamento de Medicina Comunitária, Teresina, PI, Brasil.; 3 Instituto de Pesquisa Econômica Aplicada, Diretoria de Estudos e Políticas Sociais, Brasília, DF, Brasil

This special issue focuses on releasing articles on the diagnosis, treatment, and surveillance of sexually transmitted infections (STI), based on the 2020 Clinical Protocol and Therapeutic Guidelines (PCDT) for comprehensive care for people with STI^1^. This is a joint publication by *Epidemiologia e Serviços de Saúde: revista do Sistema Único de Saúde do Brasil* [Epidemiology and Health Services: Journal of the Brazilian National Health System] (RESS) and *Revista da Sociedade Brasileira de Medicina Tropical* [Journal of the Brazilian Society of Tropical Medicine] (RSBMT), under the partnership between the two journals. It is simultaneously published in three languages - Portuguese, Spanish, and English - to promote content for healthcare professionals working in care for people with STI.

The health care for people with STI - as well as other health conditions - must reflect the best scientific evidence possible, in conjunction with indissociable contextual factors: professional experience, individual characteristics, and the health system’s capability. The systematization of the diagnosis and treatment of people with STI requires appropriated support. The recommendations should be organized and accessible to the healthcare professionals, and they should receive training and have adequate working conditions, allowing people to take care of other people.

The Brazilian National Health System (SUS), which since its creation is grounded on scientific knowledge and worldwide acknowledged best practice, promotes the development of the PCDT since the institution of the National Committee for Health Technology Incorporation in the Unified Health System in 2011. The PCDT development aims at guiding clinical practice for specific conditions through the definition of research question, search and assessment of scientific evidence, grading of certainty of evidence and strength of recommendations, writing, and public consultation, with its periodical update being provided^2^. Different documents guiding the clinical practice at SUS are previous to such methodology. Adherence to the method depends on institutional transition, as the procedure includes both the scientific rigor, the clinical expertise, and experience set in the country.

The procedure for developing the recommendations grounded on scientific evidence also depends on skilled human capital for locating and interpreting the evidence and well-conducted scientific research, free from conflict of interest, which correctly answer the clinical practice questions^3^. The use of such evidence, in its turn, requires its promotion among healthcare professionals. The adherence to the recommended practice deploys education and service supervision initiatives, among other implementation strategies^4^. Since the development of the recommendations grounded on scientific evidence up to its incorporation, barriers limit the full adherence. Its adoption undergoes culture changes that values science and healthcare professionals. Situations lacking evidence may be prioritized for SUS research, a signaling role that PCDT plays in such cases^2^. The practice of developing and implementing evidence-based recommendations enables this culture to be established, with feedback for a virtuous learning cycle. 

The 18 articles published in this special issue result from a successful partnership between RSBMT and RESS, and from the fundamental collaboration by the group of specialists encompassed, which took part in preparation and review of the manuscripts. Through this publication, RESS and RSBMT contribute to promoting the PCDT for comprehensive care for people with STI, aiming to reach different actors of SUS in all its capillarity.
